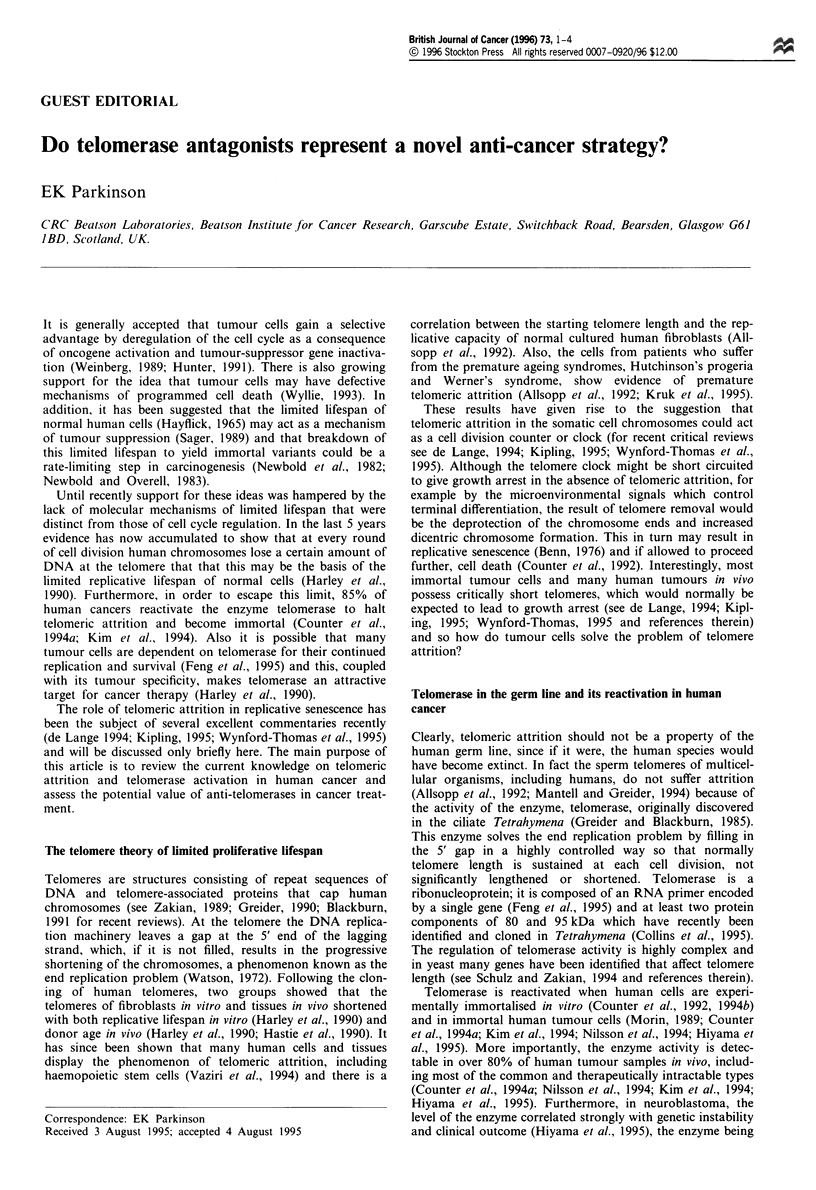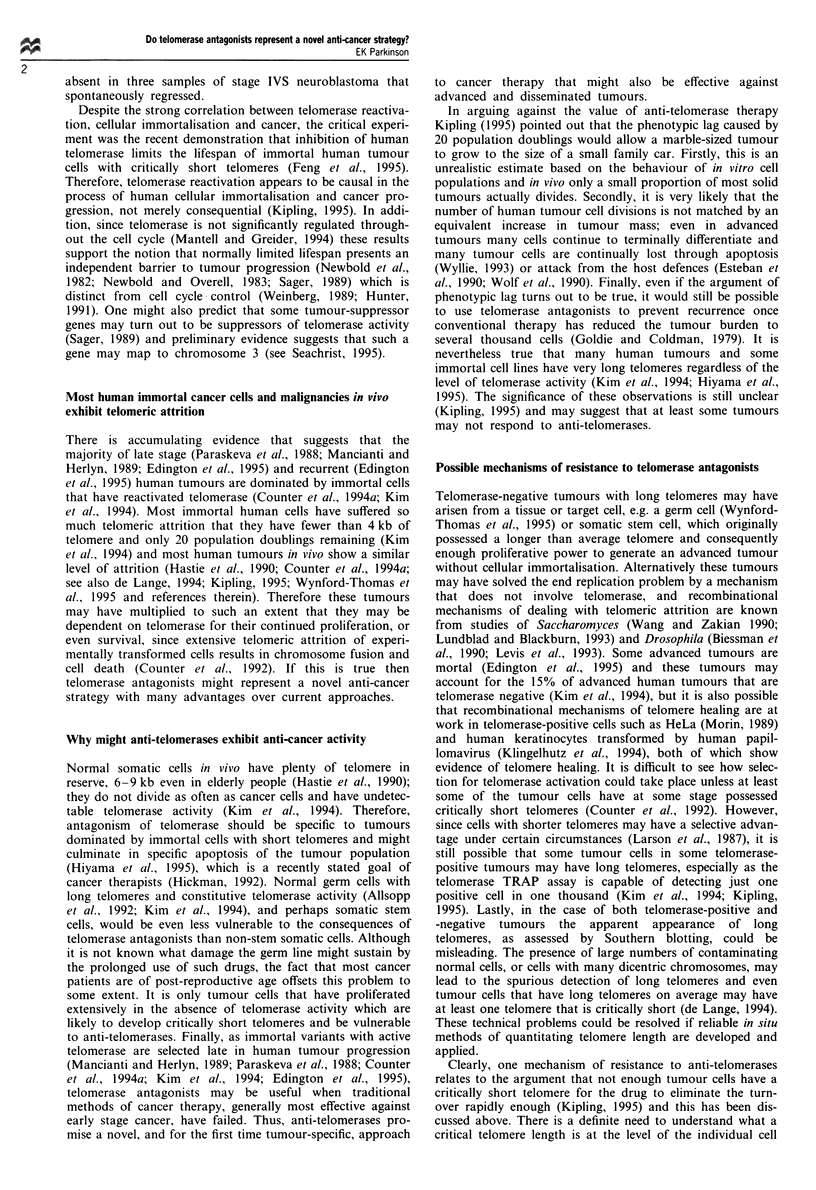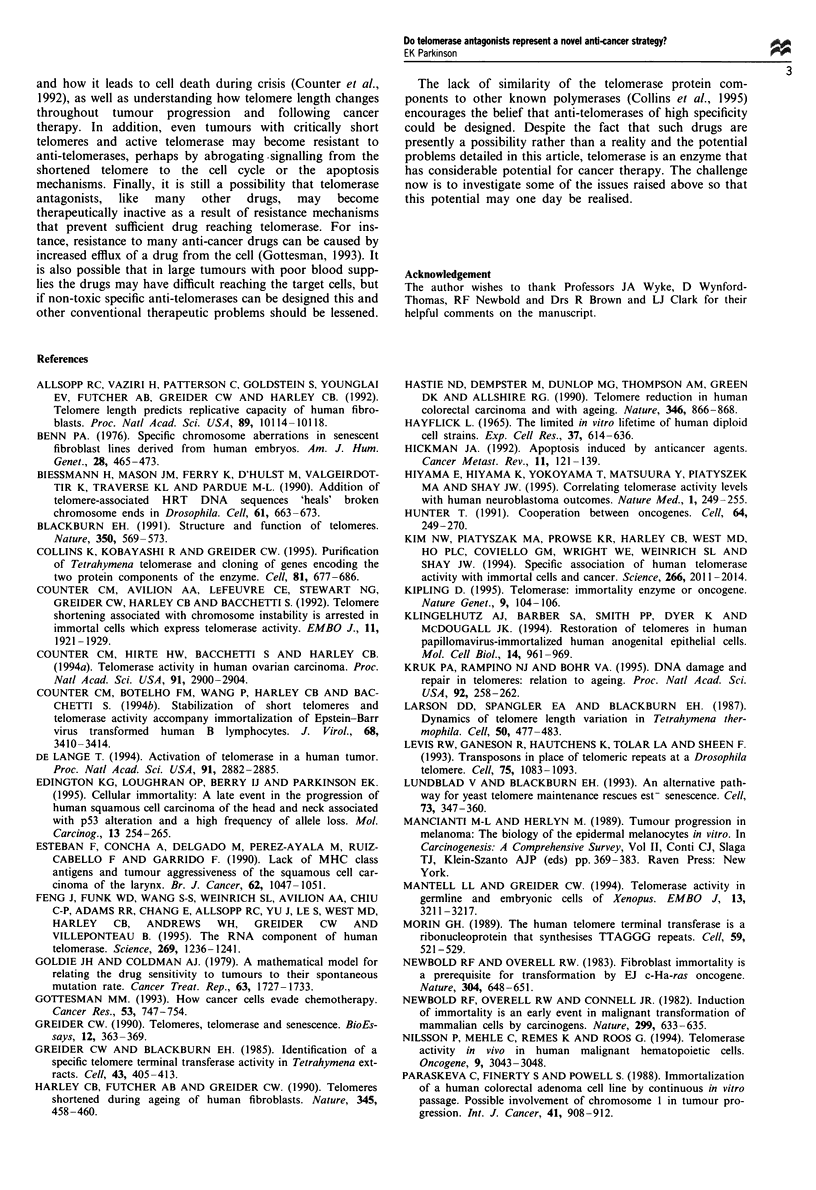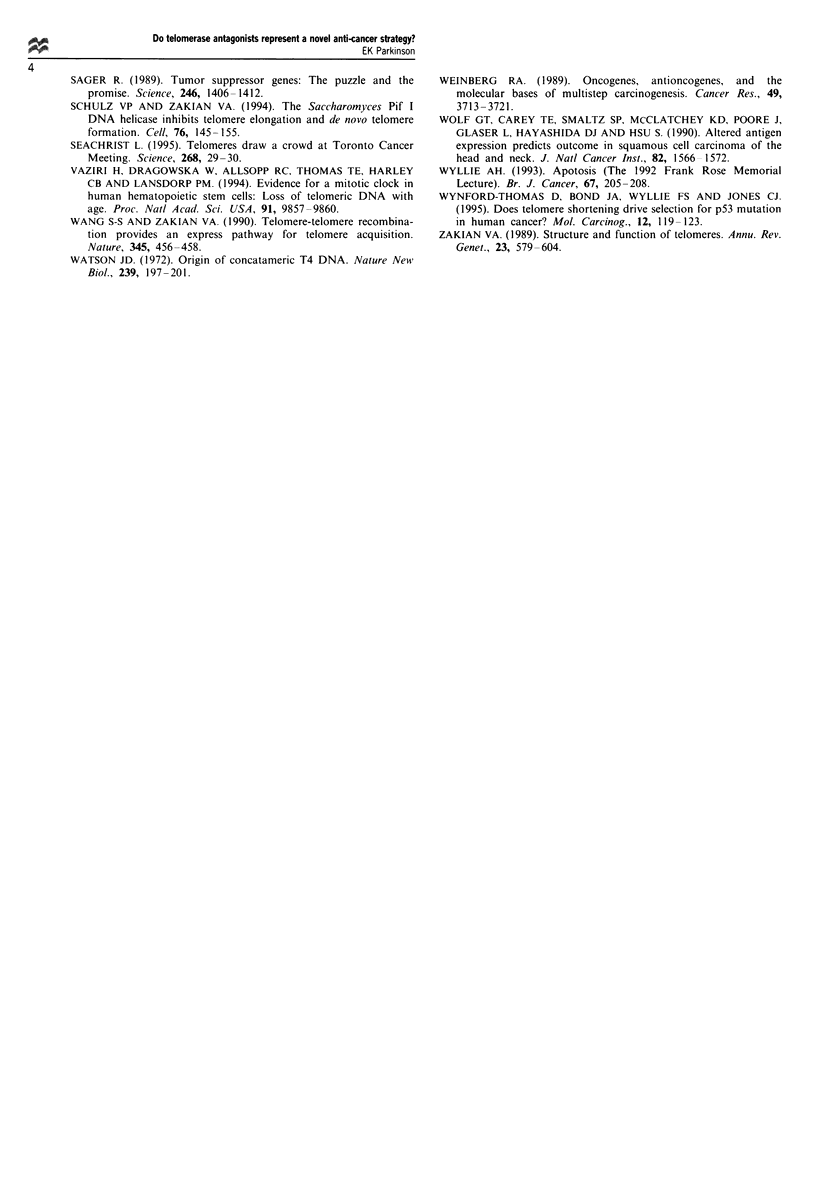# Do telomerase antagonists represent a novel anti-cancer strategy?

**DOI:** 10.1038/bjc.1996.1

**Published:** 1996-01

**Authors:** E. K. Parkinson


British Journal of Cancer (1996) 73, 1-4
© 1996 Stockton Press All rights reserved 0007-0920/96 $12.00

GUEST EDITORIAL

### Do telomerase antagonists represent a novel anti-cancer strategy?

EK Parkinson

*CRC Beatson Laboratories, Beatson Institute for Cancer Research, Garscube Estate, Switchback Road, Bearsden, Glasgow G61 1BD, Scotland, UK.*

It is generally accepted that tumour cells gain a selective advantage by deregulation of the cell cycle as a consequence of oncogene activation and tumour-suppressor gene inactivation (Weinberg, 1989; Hunter, 1991). There is also growing support for the idea that tumour cells may have defective mechanisms of programmed cell death (Wyllie, 1993). In addition, it has been suggested that the limited lifespan of normal human cells (Hayflick, 1965) may act as a mechanism of tumour suppression (Sager, 1989) and that breakdown of this limited lifespan to yield immortal variants could be a rate-limiting step in carcinogenesis (Newbold *et al.*, 1982; Newbold and Overell, 1983).

Until recently support for these ideas was hampered by the lack of molecular mechanisms of limited lifespan that were distinct from those of cell cycle regulation. In the last 5 years evidence has now accumulated to show that at every round of cell division human chromosomes lose a certain amount of DNA at the telomere that that this may be the basis of the limited replicative lifespan of normal cells (Harley *et al.*, 1990). Furthermore, in order to escape this limit, 85% of human cancers reactivate the enzyme telomerase to halt telomeric attrition and become immortal (Counter *et al.*, 1994*a*; Kim *et al.*, 1994). Also it is possible that many tumour cells are dependent on telomerase for their continued replication and survival (Feng *et al.*, 1995) and this, coupled with its tumour specificity, makes telomerase an attractive target for cancer therapy (Harley *et al.*, 1990).

The role of telomeric attrition in replicative senescence has been the subject of several excellent commentaries recently (de Lange 1994; Kipling, 1995; Wynford-Thomas *et al.*, 1995) and will be discussed only briefly here. The main purpose of this article is to review the current knowledge on telomeric attrition and telomerase activation in human cancer and assess the potential value of anti-telomerases in cancer treatment.

### The telomere theory of limited proliferative lifespan

Telomeres are structures consisting of repeat sequences of DNA and telomere-associated proteins that cap human chromosomes (see Zakian, 1989; Greider, 1990; Blackburn, 1991 for recent reviews). At the telomere the DNA replication machinery leaves a gap at the 5′ end of the lagging strand, which, if it is not filled, results in the progressive shortening of the chromosomes, a phenomenon known as the end replication problem (Watson, 1972). Following the cloning of human telomeres, two groups showed that the telomeres of fibroblasts *in vitro* and tissues *in vivo* shortened with both replicative lifespan *in vitro* (Harley *et al.*, 1990) and donor age *in vivo* (Harley *et al.*, 1990; Hastie *et al.*, 1990). It has since been shown that many human cells and tissues display the phenomenon of telomeric attrition, including haemopoietic stem cells (Vaziri *et al.*, 1994) and there is a correlation between the starting telomere length and the replicative capacity of normal cultured human fibroblasts (Allsopp *et al.*, 1992). Also, the cells from patients who suffer from the premature ageing syndromes, Hutchinson's progeria and Werner's syndrome, show evidence of premature telomeric attrition (Allsopp *et al.*, 1992; Kruk *et al.*, 1995).

These results have given rise to the suggestion that telomeric attrition in the somatic cell chromosomes could act as a cell division counter or clock (for recent critical reviews see de Lange, 1994; Kipling, 1995; Wynford-Thomas *et al.*, 1995). Although the telomere clock might be short circuited to give growth arrest in the absence of telomeric attrition, for example by the microenvironmental signals which control terminal differentiation, the result of telomere removal would be the deprotection of the chromosome ends and increased dicentric chromosome formation. This in turn may result in replicative senescence (Benn, 1976) and if allowed to proceed further, cell death (Counter *et al.*, 1992). Interestingly, most immortal tumour cells and many human tumours *in vivo* possess critically short telomeres, which would normally be expected to lead to growth arrest (see de Lange, 1994; Kipling, 1995; Wynford-Thomas, 1995 and references therein) and so how do tumour cells solve the problem of telomere attrition?

### Telomerase in the germ line and its reactivation in human cancer

Clearly, telomeric attrition should not be a property of the human germ line, since if it were, the human species would have become extinct. In fact the sperm telomeres of multicellular organisms, including humans, do not suffer attrition (Allsopp *et al.*, 1992; Mantell and Greider, 1994) because of the activity of the enzyme, telomerase, originally discovered in the ciliate *Tetrahymena* (Greider and Blackburn, 1985). This enzyme solves the end replication problem by filling in the 5′ gap in a highly controlled way so that normally telomere length is sustained at each cell division, not significantly lengthened or shortened. Telomerase is a ribonucleoprotein; it is composed of an RNA primer encoded by a single gene (Feng *et al.*, 1995) and at least two protein components of 80 and 95 kDa which have recently been identified and cloned in *Tetrahymena* (Collins *et al.*, 1995). The regulation of telomerase activity is highly complex and in yeast many genes have been identified that affect telomere length (see Schulz and Zakian, 1994 and references therein).

Telomerase is reactivated when human cells are experimentally immortalised *in vitro* (Counter *et al.*, 1992, 1994*b*) and in immortal human tumour cells (Morin, 1989; Counter *et al.*, 1994*a*; Kim *et al.*, 1994; Nilsson *et al.*, 1994; Hiyama *et al.*, 1995). More importantly, the enzyme activity is detectable in over 80% of human tumour samples *in vivo*, including most of the common and therapeutically intractable types (Counter *et al.*, 1994*a*; Nilsson *et al.*, 1994; Kim *et al.*, 1994; Hiyama *et al.*, 1995). Furthermore, in neuroblastoma, the level of the enzyme correlated strongly with genetic instability and clinical outcome (Hiyama *et al.*, 1995), the enzyme being

Correspondence: EK Parkinson
Received 3 August 1995; accepted 4 August 1995

absent in three samples of stage IVS neuroblastoma that spontaneously regressed.

Despite the strong correlation between telomerase reactivation, cellular immortalisation and cancer, the critical experiment was the recent demonstration that inhibition of human telomerase limits the lifespan of immortal human tumour cells with critically short telomeres (Feng *et al*., 1995). Therefore, telomerase reactivation appears to be causal in the process of human cellular immortalisation and cancer progression, not merely consequential (Kipling, 1995). In addition, since telomerase is not significantly regulated throughout the cell cycle (Mantell and Greider, 1994) these results support the notion that normally limited lifespan presents an independent barrier to tumour progression (Newbold *et al*., 1982; Newbold and Overell, 1983; Sager, 1989) which is distinct from cell cycle control (Weinberg, 1989; Hunter, 1991). One might also predict that some tumour-suppressor genes may turn out to be suppressors of telomerase activity (Sager, 1989) and preliminary evidence suggests that such a gene may map to chromosome 3 (see Seachrist, 1995).

### Most human immortal cancer cells and malignancies *in vivo* exhibit telomeric attrition

There is accumulating evidence that suggests that the majority of late stage (Paraskeva *et al*., 1988; Mancianti and Herlyn, 1989; Edington *et al*., 1995) and recurrent (Edington *et al*., 1995) human tumours are dominated by immortal cells that have reactivated telomerase (Counter *et al*., 1994*a*; Kim *et al*., 1994). Most immortal human cells have suffered so much telomeric attrition that they have fewer than 4 kb of telomere and only 20 population doublings remaining (Kim *et al*., 1994) and most human tumours *in vivo* show a similar level of attrition (Hastie *et al*., 1990; Counter *et al*., 1994*a*; see also de Lange, 1994; Kipling, 1995; Wynford-Thomas *et al*., 1995 and references therein). Therefore these tumours may have multiplied to such an extent that they may be dependent on telomerase for their continued proliferation, or even survival, since extensive telomeric attrition of experimentally transformed cells results in chromosome fusion and cell death (Counter *et al*., 1992). If this is true then telomerase antagonists might represent a novel anti-cancer strategy with many advantages over current approaches.

### Why might anti-telomerases exhibit anti-cancer activity

Normal somatic cells *in vivo* have plenty of telomere in reserve, 6–9 kb even in elderly people (Hastie *et al*., 1990); they do not divide as often as cancer cells and have undetectable telomerase activity (Kim *et al*., 1994). Therefore, antagonism of telomerase should be specific to tumours dominated by immortal cells with short telomeres and might culminate in specific apoptosis of the tumour population (Hiyama *et al*., 1995), which is a recently stated goal of cancer therapists (Hickman, 1992). Normal germ cells with long telomeres and constitutive telomerase activity (Allsopp *et al*., 1992; Kim *et al*., 1994), and perhaps somatic stem cells, would be even less vulnerable to the consequences of telomerase antagonists than non-stem somatic cells. Although it is not known what damage the germ line might sustain by the prolonged use of such drugs, the fact that most cancer patients are of post-reproductive age offsets this problem to some extent. It is only tumour cells that have proliferated extensively in the absence of telomerase activity which are likely to develop critically short telomeres and be vulnerable to anti-telomerases. Finally, as immortal variants with active telomerase are selected late in human tumour progression (Mancianti and Herlyn, 1989; Paraskeva *et al*., 1988; Counter *et al*., 1994*a*; Kim *et al*., 1994; Edington *et al*., 1995), telomerase antagonists may be useful when traditional methods of cancer therapy, generally most effective against early stage cancer, have failed. Thus, anti-telomerases promise a novel, and for the first time tumour-specific, approach to cancer therapy that might also be effective against advanced and disseminated tumours.

In arguing against the value of anti-telomerase therapy Kipling (1995) pointed out that the phenotypic lag caused by 20 population doublings would allow a marble-sized tumour to grow to the size of a small family car. Firstly, this is an unrealistic estimate based on the behaviour of *in vitro* cell populations and *in vivo* only a small proportion of most solid tumours actually divides. Secondly, it is very likely that the number of human tumour cell divisions is not matched by an equivalent increase in tumour mass; even in advanced tumours many cells continue to terminally differentiate and many tumour cells are continually lost through apoptosis (Wyllie, 1993) or attack from the host defences (Esteban *et al*., 1990; Wolf *et al*., 1990). Finally, even if the argument of phenotypic lag turns out to be true, it would still be possible to use telomerase antagonists to prevent recurrence once conventional therapy has reduced the tumour burden to several thousand cells (Goldie and Coldman, 1979). It is nevertheless true that many human tumours and some immortal cell lines have very long telomeres regardless of the level of telomerase activity (Kim *et al*., 1994; Hiyama *et al*., 1995). The significance of these observations is still unclear (Kipling, 1995) and may suggest that at least some tumours may not respond to anti-telomerases.

### Possible mechanisms of resistance to telomerase antagonists

Telomerase-negative tumours with long telomeres may have arisen from a tissue or target cell, e.g. a germ cell (Wynford-Thomas *et al*., 1995) or somatic stem cell, which originally possessed a longer than average telomere and consequently enough proliferative power to generate an advanced tumour without cellular immortalisation. Alternatively these tumours may have solved the end replication problem by a mechanism that does not involve telomerase, and recombinational mechanisms of dealing with telomeric attrition are known from studies of *Saccharomyces* (Wang and Zakian 1990; Lundblad and Blackburn, 1993) and *Drosophila* (Biessman *et al*., 1990; Levis *et al*., 1993). Some advanced tumours are mortal (Edington *et al*., 1995) and these tumours may account for the 15% of advanced human tumours that are telomerase negative (Kim *et al*., 1994), but it is also possible that recombinational mechanisms of telomere healing are at work in telomerase-positive cells such as HeLa (Morin, 1989) and human keratinocytes transformed by human papillomavirus (Klingelhutz *et al*., 1994), both of which show evidence of telomere healing. It is difficult to see how selection for telomerase activation could take place unless at least some of the tumour cells have at some stage possessed critically short telomeres (Counter *et al*., 1992). However, since cells with shorter telomeres may have a selective advantage under certain circumstances (Larson *et al*., 1987), it is still possible that some tumour cells in some telomerase-positive tumours may have long telomeres, especially as the telomerase TRAP assay is capable of detecting just one positive cell in one thousand (Kim *et al*., 1994; Kipling, 1995). Lastly, in the case of both telomerase-positive and -negative tumours the apparent appearance of long telomeres, as assessed by Southern blotting, could be misleading. The presence of large numbers of contaminating normal cells, or cells with many dicentric chromosomes, may lead to the spurious detection of long telomeres and even tumour cells that have long telomeres on average may have at least one telomere that is critically short (de Lange, 1994). These technical problems could be resolved if reliable *in situ* methods of quantitating telomere length are developed and applied.

Clearly, one mechanism of resistance to anti-telomerases relates to the argument that not enough tumour cells have a critically short telomere for the drug to eliminate the turnover rapidly enough (Kipling, 1995) and this has been discussed above. There is a definite need to understand what a critical telomere length is at the level of the individual cell and how it leads to cell death during crisis (Counter *et al.*, 1992), as well as understanding how telomere length changes throughout tumour progression and following cancer therapy. In addition, even tumours with critically short telomeres and active telomerase may become resistant to anti-telomerases, perhaps by abrogating signalling from the shortened telomere to the cell cycle or the apoptosis mechanisms. Finally, it is still a possibility that telomerase antagonists, like many other drugs, may become therapeutically inactive as a result of resistance mechanisms that prevent sufficient drug reaching telomerase. For instance, resistance to many anti-cancer drugs can be caused by increased efflux of a drug from the cell (Gottesman, 1993). It is also possible that in large tumours with poor blood supplies the drugs may have difficult reaching the target cells, but if non-toxic specific anti-telomerases can be designed this and other conventional therapeutic problems should be lessened.

The lack of similarity of the telomerase protein components to other known polymerases (Collins *et al.*, 1995) encourages the belief that anti-telomerases of high specificity could be designed. Despite the fact that such drugs are presently a possibility rather than a reality and the potential problems detailed in this article, telomerase is an enzyme that has considerable potential for cancer therapy. The challenge now is to investigate some of the issues raised above so that this potential may one day be realised.

**Acknowledgement**
The author wishes to thank Professors JA Wyke, D Wynford-Thomas, RF Newbold and Drs R Brown and LJ Clark for their helpful comments on the manuscript.

**References**

ALLSOPP RC, VAZIRI H, PATTERSON C, GOLDSTEIN S, YOUNGLAI EV, FUTCHER AB, GREIDER CW AND HARLEY CB. (1992). Telomere length predicts replicative capacity of human fibroblasts. *Proc. Natl Acad. Sci. USA*, **89**, 10114–10118.

BENN PA. (1976). Specific chromosome aberrations in senescent fibroblast lines derived from human embryos. *Am. J. Hum. Genet.*, **28**, 465–473.

BIESSMANN H, MASON JM, FERRY K, D'HULST M, VALGEIRDOTTIR K, TRAVERSE KL AND PARDUE M-L. (1990). Addition of telomere-associated HRT DNA sequences 'heals' broken chromosome ends in *Drosophila*. *Cell*, **61**, 663–673.

BLACKBURN EH. (1991). Structure and function of telomeres. *Nature*, **350**, 569–573.

COLLINS K, KOBAYASHI R AND GREIDER CW. (1995). Purification of *Tetrahymena* telomerase and cloning of genes encoding the two protein components of the enzyme. *Cell*, **81**, 677–686.

COUNTER CM, AVILION AA, LEFEUVRE CE, STEWART NG, GREIDER CW, HARLEY CB AND BACCHETTI S. (1992). Telomere shortening associated with chromosome instability is arrested in immortal cells which express telomerase activity. *EMBO J.*, **11**, 1921–1929.

COUNTER CM, HIRTE HW, BACCHETTI S AND HARLEY CB. (1994*a*). Telomerase activity in human ovarian carcinoma. *Proc. Natl Acad. Sci. USA*, **91**, 2900–2904.

COUNTER CM, BOTELHO FM, WANG P, HARLEY CB AND BACCHETTI S. (1994*b*). Stabilization of short telomeres and telomerase activity accompany immortalization of Epstein–Barr virus transformed human B lymphocytes. *J. Virol.*, **68**, 3410–3414.

DE LANGE T. (1994). Activation of telomerase in a human tumor. *Proc. Natl Acad. Sci. USA*, **91**, 2882–2885.

EDINGTON KG, LOUGHRAN OP, BERRY IJ AND PARKINSON EK. (1995). Cellular immortality: A late event in the progression of human squamous cell carcinoma of the head and neck associated with p53 alteration and a high frequency of allele loss. *Mol. Carcinog.*, **13** 254–265.

ESTEBAN F, CONCHA A, DELGADO M, PEREZ-AYALA M, RUIZ-CABELLO F AND GARRIDO F. (1990). Lack of MHC class antigens and tumour aggressiveness of the squamous cell carcinoma of the larynx. *Br. J. Cancer*, **62**, 1047–1051.

FENG J, FUNK WD, WANG S-S, WEINRICH SL, AVILION AA, CHIU C-P, ADAMS RR, CHANG E, ALLSOPP RC, YU J, LE S, WEST MD, HARLEY CB, ANDREWS WH, GREIDER CW AND VILLEPONTEAU B. (1995). The RNA component of human telomerase. *Science*, **269**, 1236–1241.

GOLDIE JH AND COLDMAN AJ. (1979). A mathematical model for relating the drug sensitivity to tumours to their spontaneous mutation rate. *Cancer Treat. Rep.*, **63**, 1727–1733.

GOTTESMAN MM. (1993). How cancer cells evade chemotherapy. *Cancer Res.*, **53**, 747–754.

GREIDER CW. (1990). Telomeres, telomerase and senescence. *BioEssays*, **12**, 363–369.

GREIDER CW AND BLACKBURN EH. (1985). Identification of a specific telomere terminal transferase activity in *Tetrahymena* extracts. *Cell*, **43**, 405–413.

HARLEY CB, FUTCHER AB AND GREIDER CW. (1990). Telomeres shortened during ageing of human fibroblasts. *Nature*, **345**, 458–460.

HASTIE ND, DEMPSTER M, DUNLOP MG, THOMPSON AM, GREEN DK AND ALLSHIRE RG. (1990). Telomere reduction in human colorectal carcinoma and with ageing. *Nature*, **346**, 866–868.

HAYFLICK L. (1965). The limited *in vitro* lifetime of human diploid cell strains. *Exp. Cell Res.*, **37**, 614–636.

HICKMAN JA. (1992). Apoptosis induced by anticancer agents. *Cancer Metast. Rev.*, **11**, 121–139.

HIYAMA E, HIYAMA K, YOKOYAMA T, MATSUURA Y, PIATYSZEK MA AND SHAY JW. (1995). Correlating telomerase activity levels with human neuroblastoma outcomes. *Nature Med.*, **1**, 249–255.

HUNTER T. (1991). Cooperation between oncogenes. *Cell*, **64**, 249–270.

KIM NW, PIATYSZAK MA, PROWSE KR, HARLEY CB, WEST MD, HO PLC, COVIELLO GM, WRIGHT WE, WEINRICH SL AND SHAY JW. (1994). Specific association of human telomerase activity with immortal cells and cancer. *Science*, **266**, 2011–2014.

KIPLING D. (1995). Telomerase: immortality enzyme or oncogene. *Nature Genet.*, **9**, 104–106.

KLINGELHUTZ AJ, BARBER SA, SMITH PP, DYER K AND MCDOUGALL JK. (1994). Restoration of telomeres in human papillomavirus-immortalized human anogenital epithelial cells. *Mol. Cell Biol.*, **14**, 961–969.

KRUK PA, RAMPINO NJ AND BOHR VA. (1995). DNA damage and repair in telomeres: relation to ageing. *Proc. Natl Acad. Sci. USA*, **92**, 258–262.

LARSON DD, SPANGLER EA AND BLACKBURN EH. (1987). Dynamics of telomere length variation in *Tetrahymena thermophila*. *Cell*, **50**, 477–483.

LEVIS RW, GANESON R, HAUTCHENS K, TOLAR LA AND SHEEN F. (1993). Transposons in place of telomeric repeats at a *Drosophila* telomere. *Cell*, **75**, 1083–1093.

LUNDBLAD V AND BLACKBURN EH. (1993). An alternative pathway for yeast telomere maintenance rescues est$^-$ senescence. *Cell*, **73**, 347–360.

MANCIANTI M-L AND HERLYN M. (1989). Tumour progression in melanoma: The biology of the epidermal melanocytes *in vitro*. In *Carcinogenesis: A Comprehensive Survey*, Vol II, Conti CJ, Slaga TJ, Klein-Szanto AJP (eds) pp. 369–383. Raven Press: New York.

MANTELL LL AND GREIDER CW. (1994). Telomerase activity in germline and embryonic cells of *Xenopus*. *EMBO J*, **13**, 3211–3217.

MORIN GH. (1989). The human telomere terminal transferase is a ribonucleoprotein that synthesises TTAGGG repeats. *Cell*, **59**, 521–529.

NEWBOLD RF AND OVERELL RW. (1983). Fibroblast immortality is a prerequisite for transformation by EJ c-Ha-*ras* oncogene. *Nature*, **304**, 648–651.

NEWBOLD RF, OVERELL RW AND CONNELL JR. (1982). Induction of immortality is an early event in malignant transformation of mammalian cells by carcinogens. *Nature*, **299**, 633–635.

NILSSON P, MEHLE C, REMES K AND ROOS G. (1994). Telomerase activity *in vivo* in human malignant hematopoietic cells. *Oncogene*, **9**, 3043–3048.

PARASKEVA C, FINERTY S AND POWELL S. (1988). Immortalization of a human colorectal adenoma cell line by continuous *in vitro* passage. Possible involvement of chromosome 1 in tumour progression. *Int. J. Cancer*, **41**, 908–912.

SAGER R. (1989). Tumor suppressor genes: The puzzle and the promise. *Science*, **246**, 1406–1412.
SCHULZ VP AND ZAKIAN VA. (1994). The *Saccharomyces* Pif I DNA helicase inhibits telomere elongation and *de novo* telomere formation. *Cell*, **76**, 145–155.
SEACHRIST L. (1995). Telomeres draw a crowd at Toronto Cancer Meeting. *Science*, **268**, 29–30.
VAZIRI H, DRAGOWSKA W, ALLSOPP RC, THOMAS TE, HARLEY CB AND LANSDORP PM. (1994). Evidence for a mitotic clock in human hematopoietic stem cells: Loss of telomeric DNA with age. *Proc. Natl Acad. Sci. USA*, **91**, 9857–9860.
WANG S-S AND ZAKIAN VA. (1990). Telomere-telomere recombination provides an express pathway for telomere acquisition. *Nature*, **345**, 456–458.
WATSON JD. (1972). Origin of concatameric T4 DNA. *Nature New Biol.*, **239**, 197–201.
WEINBERG RA. (1989). Oncogenes, antioncogenes, and the molecular bases of multistep carcinogenesis. *Cancer Res.*, **49**, 3713–3721.
WOLF GT, CAREY TE, SMALTZ SP, McCLATCHEY KD, POORE J, GLASER L, HAYASHIDA DJ AND HSU S. (1990). Altered antigen expression predicts outcome in squamous cell carcinoma of the head and neck. *J. Natl Cancer Inst.*, **82**, 1566–1572.
WYLLIE AH. (1993). Apotosis (The 1992 Frank Rose Memorial Lecture). *Br. J. Cancer*, **67**, 205–208.
WYNFORD-THOMAS D, BOND JA, WYLLIE FS AND JONES CJ. (1995). Does telomere shortening drive selection for p53 mutation in human cancer? *Mol. Carcinog.*, **12**, 119–123.
ZAKIAN VA. (1989). Structure and function of telomeres. *Annu. Rev. Genet.*, **23**, 579–604.